# Pesticide regulatory risk assessment, monitoring, and fate studies in the northern zone: recommendations from a Nordic-Baltic workshop

**DOI:** 10.1007/s11356-016-7087-1

**Published:** 2016-06-22

**Authors:** Marianne Stenrød, Marit Almvik, Ole Martin Eklo, Anne Louise Gimsing, Roger Holten, Kai Künnis-Beres, Mats Larsbo, Linas Putelis, Katri Siimes, Inara Turka, Jaana Uusi-Kämppä

**Affiliations:** Norwegian Institute of Bioeconomy Research (NIBIO), Høgskoleveien 7, NO-1430 Ås, Norway; Ministry of Environment and Food of Denmark, Environmental Protection Agency, Strandgade 29, DK-1401 København K, Denmark; Norwegian Food Safety Authority, P.O. Box 383, NO-2381 Brumunddal, Norway; Institute of Marine Systems, Tallinn University of Technology, Ehitajate tee 5, 19086 Tallinn, Estonia; National Institute of Chemical Physics and Biophysics, Akadeemia Tee 23, EE-12618 Tallinn, Estonia; Department of Soil and Environment, Swedish University of Agricultural Sciences (SLU), P.O. Box 7014, SE-75007 Uppsala, Sweden; Lithuanian Research Centre for Agriculture and Forestry, Instituto aleja 1, Akademija, LT-58344 Kėdainiai District, Lithuania; Finnish Environment Institute (SYKE), P.O. Box 140, FI-00251 Helsinki, Finland; Latvia University of Agriculture, 2 Liela Street, Jelgava, LV-3001 Latvia; Natural Resources Institute Finland (LUKE), Viikinkaari 4, FI-00790 Helsinki, Finland

## Introduction

**Fig. 1:**
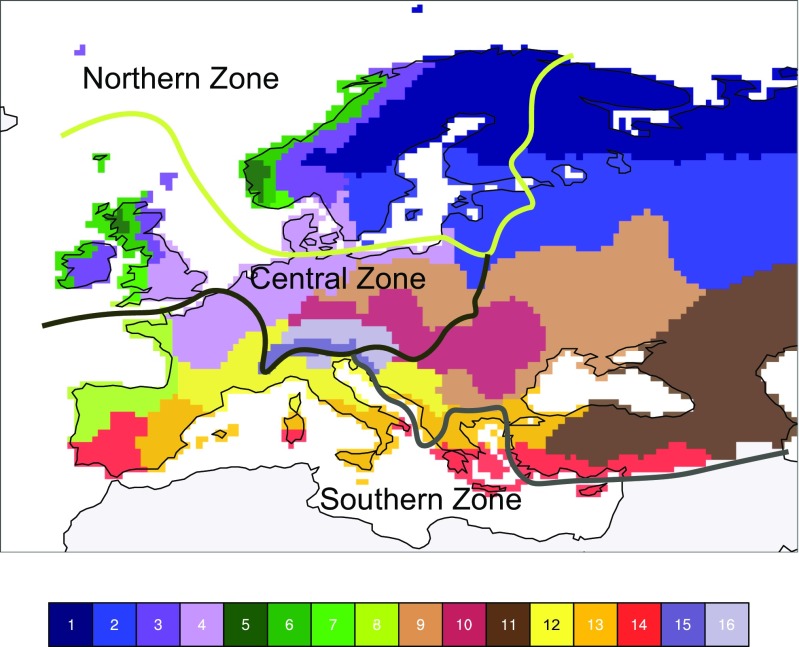
Zones for pesticide authorization overlaid on climatic zones for pesticide modeling (reprinted from Blenkinsop et al. 2008 with permission from Elsevier) in Europe

The recent revision of the legal framework for authorization of use of plant protection products and pesticides within the European Union/European Economic Area (EU/EEA; Regulation EC 1107/2009, Directive 2009/128/EC) imposes a need for close collaboration across country borders within the three pesticide authorization zones (designated the north, central, and south zones) in Europe. The principles of zonal evaluation and mutual recognition embedded in Regulation EC 1107/2009 concerning marketing of plant protection products are intended to reduce the approval times for pesticides. However, the three authorization zones represent a very simplified view compared to the 16 climatic zones/scenarios that have been outlined for pesticide modeling in Europe (Blenkinsop et al. 2008; Fig. 1). Pedoclimatic or agricultural constraints could entitle the individual states to adopt restrictions on the use of pesticides approved within their zone or even to refuse approval.

To achieve a sound scientific basis for zonal evaluation and collaboration on a regulatory level, it is also necessary to increase research collaboration and knowledge exchange within the scientific community. Here, we report the main conclusions and recommendations from a Nordic-Baltic workshop on the environmental fate of pesticides, which was conducted in Ås, Norway, in September 2014 with the aim of promoting knowledge exchange, network building, and a common agenda for future research within the northern zone.

### Pesticide regulatory risk assessment in the northern zone

#### Zonal evaluation and mutual recognition

The “Guidance document on work sharing in the Northern zone in the authorization of plant protection products” (Anonymous 2015) states that the northern zone cooperation includes the EU member states Denmark, Sweden, Finland, Estonia, Latvia, and Lithuania, as well as the European Economic Community/European Free Trade Association (EEC/EFTA) members Norway and Iceland. The guidance document was implemented in all countries within the zone from January 2015. Climatic zones for pesticide modeling (Blenkinsop et al. 2008) reflect the complexity of the different authorization zones within Europe (Fig. 1). According to this classification, the northern zone countries cover seven of the 16 climatic zones (Table 1). The variation within the northern zone is further illustrated by the 13 environmental zones representing an aggregation of the environmental stratification of Europe (Metzger et al. 2005; Jongman et al. 2006), five of which are covered by the northern zone countries (Table 2). The duration of the growing season and the sum of active temperatures are doubled when moving southward from the alpine north to the Atlantic north. This will inevitably affect the possibility of harmonizing risk assessment procedures and/or requirements between the countries within the northern zone, and it will also influence the commercial viability of the pesticide industry.

**Table 1:** Climate zones for pesticide modeling (Blenkinsop et al. 2008) in the northern zone countries

Climate zone	Climate characteristics
2	Temperate maritime
4	North European and continental, cool and dry
7	Modified upland temperate maritime, more frequent extremes
10	North European, cold and dry
12	Very wet and mountainous maritime, more frequent extremes
13	Wet and maritime on exposed western coasts, more frequent extremes
16	Modified temperate maritime, cool with moderate precipitation

**Table 2:** Growing season characteristics in the northern zone based on the environmental stratification of Europe (Metzger et al. 2005; Jongman et al. 2006)

Environmental zone	Growing season characteristics	
	Duration	Sum of active temperatures
	(days (min–max))	(>+10 °C (min–max))
Alpine North (ALN)	130 (116–155)	1416 (1277–1719)
Boreal (BOR)	157 (126–185)	1966 (1471–2523)
Nemoral (NEM)	196 (190–204)	2717 (2561–2898)
Continental (CON)	227 (213–257)	3294 (3037–3049)^a^
Atlantic North (ATN)	255 (199–278)	3198 (2459–3214)

^a^The highest values are reached in the continental parts of the Balkan Peninsula

Due to the strict limits of the timeline for the zonal evaluation (SANCO/13169/2010 rev. 9), there must be good agreement between the countries in the northern zone to ensure a satisfactory risk assessment. The time frame during which the member states are to appraise specific national concerns comprises a period of 6 weeks for commenting on the draft regulatory report and 120 days for assessment after the initial zonal evaluation.

#### EU scenarios and model performance in the northern zone countries

Several studies have been aimed at harmonizing and simplifying the requirements for groundwater risk assessment within the northern zone. Considering leaching to groundwater, a comparison including 11 different scenarios and using the models PEARL, PELMO, and MACROinFOCUS showed substantial disparities between countries (Stenemo and Lousa Alvin 2013). However, the results of that evaluation have also revealed large differences between the models and scenarios used within the EU, and only a certain degree of harmonization has been achieved in the northern zone collaboration (Anonymous 2015). The modeling that is required in Sweden is now also considered sufficient in Norway, and the modeling required in Denmark (which entails more thorough assessment of risks related to groundwater because it represents an important source of drinking water) is considered sufficient in Lithuania. In a recent study (Burns et al. 2015), the MACRO model was used to assess the overall representativeness and protectiveness of the national and FOCUS groundwater approaches for modeling the northern zone. The results showed good protectiveness in general, but the main conclusion was that all the national scenarios for the northern zone countries should be included in regulatory simulations.

Compared to the rest of Europe, the northern part of Scandinavia is faced with specific challenges that are associated with different dominating soil types and a cold climate with freezing and thawing of soil during winter. A pilot study employing the MACRO model to compare the FOCUS SWASH scenarios and the Norwegian scenarios in WISPE indicated that the choice of endpoints has a substantial impact on the simulation results (VKM 2015). In that investigation, discrepancies between the scenarios were small with EU endpoints but were large with Norwegian endpoints (cold climate and young soils). Defined according to FOCUS (2001), the temperature conditions for most of Norway fall into the worst-case (6.6–10 °C) or extreme worst-case (<6 °C) categories, and the precipitation and recharge are chiefly in the extreme worst-case category (>300 mm average annual recharge, >1000 mm average annual rainfall).

In Sweden, the MACRO-SE model has been used for regional-scale assessment of the impact of climate change on pesticide leaching to groundwater, considering both direct effects (increased temperatures and changes in precipitation patterns) and indirect effects (e.g., related to altered land use and crop protection needs) (Steffens et al. 2015). This regionalized version of the MACRO model allows complete parameterization based on soil maps and detailed crop statistics. Assessments using this model have suggested an increased risk under the current climate projections, but they have also highlighted both the need for sound geographic information and the challenges connected with model parameterization (Moeys et al. 2012; van den Berg et al. 2012; Vanderborght et al. 2011), as well as the large uncertainty arising from the climate input (Steffens et al. 2014).

Thus far, comparisons of model performance for risk of runoff to surface water have not been performed for all the northern zone countries. Moreover, the surface water exposure assessment has been identified as one of the least harmonized types of evaluation within this zone, and this is due to assumptions of low representativeness of the standard EU scenarios and to the differences between countries with regard to requirements for effects of mitigation measures (e.g., non-spray buffer zones and drift-reducing nozzles). As an example, Norway requires that the worst-case situation be assessed in all available modeling scenarios, assuming that none of the existing EU modeling scenarios will yield sufficiently conservative estimates for the mountainous Norwegian landscapes. Consequently, Norway is now using the WISPE model/application (Bolli et al. 2013) to develop national surface water scenarios that include areas with a steeper slope (approx. 13 %) than in the EU scenarios (approx. 4 %). In Finland, the slopes are seldom steep and most of the fields have subsurface drainage systems, but despite that, studies have shown higher concentrations and losses of pesticides for surface runoff than for drainage discharge (e.g., Uusi-Kämppä et al. in preparation; Laitinen 2000; Siimes et al. 2006; Laitinen et al. 2009). The results of the cited investigations suggest that surface water scenarios for regulatory risk assessment within the northern zone should simultaneously take into account both surface runoff and drainage discharge as transport routes to surface water. In addition, the northern zone countries often require assessment of the FOCUS surface water scenarios D1, and in Norway this also applies to D2 (FOCUS 2001), which represents the worst case for drain flow. Current FOCUS surface water scenarios (>10 years old) are presently undergoing revision, and new and revised scenarios from the European Food Safety Authority (EFSA) are expected within the next 1 to 2 years. Aspects of the northern zone should be included in this work to ensure that it will be possible to perform harmonized risk assessments utilizing scenarios, endpoints, and models that encompass the specific conditions prevailing in the northern zone countries.

By comparison, better harmonization is achieved for estimation of predicted environmental concentrations (PECs) in soil. It is plausible that the Finnish PEC calculator (http://www.tukes.fi/pecsoilcalculator) that is currently in use can be further developed into a northern zone PEC calculator.

### Pesticide monitoring in the northern zone countries

Targeted and country-specific pesticide monitoring programs that consider the impact of weather conditions on the risk of leaching and runoff of pesticides are a prerequisite to ensure sustainable use of pesticides (Directive 2009/128/EC). The pesticide monitoring efforts in the Nordic countries have been summarized by Fauser and Mogensen (2002) and Kreuger (2007). In Denmark, the first analysis of pesticides in groundwater was begun in the early 1980s, and in Sweden the first analysis in surface water (seven rivers in the southern part of the country) was performed in 1985. In Norway, long-term monitoring of pesticides was established in the early 1990s. The development of improved analytical techniques contributed to the initiation of monitoring programs in the Nordic countries. Today, a diverse range of pesticide monitoring programs are being conducted, including the following: pesticide leaching assessment efforts in Denmark (e.g., Brüsch et al. 2015); grab sampling of rivers, tributaries (Karjalainen et al. 2014), and groundwater wells (e.g., Vuorimaa et al. 2007) in Finland; composite sampling for pesticide monitoring in agricultural streams in Sweden (e.g., Lindström et al. 2013) and Norway (e.g., Bechmann and Deelstra 2013). Pesticide monitoring activities in the Baltic countries are more limited, although there is intensive crop production in areas with a potentially high risk of leaching (nitrate vulnerable zone) (Jansons et al. 2011).

Groundwater constitutes 100 % of the drinking water supply in Denmark, and hence the pesticide leaching assessment program is essential to secure safe use of pesticides and clean groundwater free of pesticide residues. Assessment sites are located in five separate areas that have disparate geological “origins” (i.e., soil type and origin) and also represent the different precipitation “zones” in Denmark. Extensive quality procedures have been established to evaluate the assessment results, because these findings are used in the approval process for pesticides in Denmark.

The Swedish and Norwegian long-term pesticide monitoring programs cover selected small agricultural catchments and are mainly aimed at registering cropping practices and occurrence of pesticide residues in surface water in intensively farmed areas, because the drinking water supply in these countries is largely dependent on clean surface waters. The monitoring programs in Sweden and Norway presently comprise about 130 and 115 different substances, respectively (Lindström et al. 2013, 2015; Stenrød 2015). In Finland, similar monitoring was carried out for only 1 year in a single catchment and included 98 compounds (Siimes et al. in preparation). In monitoring data on low-dose herbicides in Sweden, detection frequency was related to the treated area (Kreuger and Adielsson 2008), and monitoring results have also shown an association between the detected pesticide concentrations and application rates in this country (Lindström et al. 2013).

The long-term data series illustrate the importance of the coverage of the chemical analysis and the effect of reduced quantification limits. In contrast to grab sampling, automated continuous sampling ensures that a larger proportion of the pesticides present are detected. Flow-proportional composite sampling is used at Norwegian sampling sites, and time-proportional composite sampling is the primary method in Swedish research catchments (with grab sampling in rivers). The flow-proportional water sampling is accurate for determining total loads, but additional sampling may be necessary to study the quality of the water (e.g., Kyllmar et al. 2014). A flow-triggered sampling method has been employed in one of the Swedish monitoring catchments since 2006 to study the peak concentrations of pesticides (Bundschuh et al. 2014; Lindström et al. 2015), and the results show that automated composite sampling procedures reveal the long-term exposure pattern, whereas assessment of peak exposures requires a flow-event-triggered high-resolution sampling strategy. The environmental risk associated with the pesticide concentrations detected is assessed in relation to an environmental quality standard (EQS). Such analysis is based chiefly on a chemical-by-chemical approach, but efforts are also being made to evaluate the effects of pesticide mixtures using the toxic-unit concept (Bundschuh et al. 2014; Petersen et al. 2013, 2015).

The pesticide monitoring efforts in Estonia thus far include grab-sampling-based monitoring of selected rivers covering a small number of substances (<10) in 2003–2008 and a broader screening (47 substances) at four locations in 2010, followed by a WFD compliance study of priority substances in 19 rivers in 2011. The overall picture indicates few detections of substances in concentrations below regulatory limits (pers. comm. Künnis-Beres). Groundwater monitoring in the nitrate vulnerable zone during 2007–2011 resulted in no detections of >0.1 μg/L. Furthermore, a soil survey with sampling of 118 agricultural fields across Estonia showed in general no detections above permitted levels (pers. comm. Künnis-Beres).

In summary, the complementarity of the Nordic-Baltic monitoring systems should be exploited across borders, but there is still a need for country-specific monitoring programs to ensure that country-specific considerations and soil and weather conditions are properly addressed. Furthermore, the agricultural practices in the Nordic-Baltic countries differ markedly, being governed not only by topography and soil and weather conditions, but also by sociocultural conditions and government regulations.

### Fate of pesticides in the northern zone

#### Impact of cold winter conditions on pesticide fate

Pesticide fate research has shown that Nordic soil and climate conditions are challenging due to slow degradation and the risk of mobilization of sorbed pesticides in winter/spring caused by the freezing/thawing of soil (Almvik et al. 2014; Laitinen et al. 2006, 2009; Stenrød et al. 2008; Siimes et al. 2006). There have been some efforts within the pesticide monitoring programs in the Nordic countries to assess the loss of pesticides during the winter period. Inasmuch as the observed concentrations have been low, such measurements have been made only during a shorter period in Finland and only occasionally in Norway (Karjalainen et al. 2014; Ludvigsen and Lode 2002). Considering the climate changes entailing more intense autumn precipitation events, mild winters with frequent freeze/thaw events, and increased risk of leaching and surface runoff, there is clearly a need for new data on the risk of pesticide loss during the winter season in cold climate regions. The Swedish monitoring program has included sampling in winter at two sites over the last few years (Lindström et al. 2015), and the results obtained will help us target future efforts towards monitoring the high leaching risk periods.

#### Pesticides of particular concern in the northern zone

The countries in the northern zone have no common agenda as to what pesticides are of particular concern. A review of existing data on pesticide use, monitoring results, information on toxicity to aquatic and terrestrial organisms, and environmental fate characteristics under northern zone conditions is a prerequisite to identify common challenges within the zone. The following criteria for defining pesticides of concern were discussed at the workshop: (1) extensive use; (2) frequent detections in monitoring programs; (3) toxicity to aquatic and/or terrestrial organisms; (4) measured environmental concentrations (MECs) above (no) effect concentrations (P(N)ECs); (5) cumulative risk assessment and compounds of particular concern with regard to mixture toxicity (e.g., synergists); and (6) slow degradation in colder climates and northern soils. In addition to the traditional focus on the less sorbed and/or more mobile compounds, further attention should be given to strongly sorbed pesticides. Greater attention should also be paid to autumn-applied pesticides and pesticide metabolites in light of the challenges caused by a changing climate, such as altered precipitation patterns (e.g., more frequent heavy autumn rain) and rising temperatures (e.g., expected faster degradation rates) (Kjellström et al. 2011). Moreover, the need to apply pesticides is increasing as a result of improved overwintering conditions for current weeds and plant pests and pathogens, and also because a change in climate might create favorable conditions for new and invasive species of that type in the northern zone (e.g., Saikkonen et al. 2012). Kattwinkel et al. (2011) simulated potential exposure to insecticides in 25 EU countries under the climate conditions in 1990 and 2090, and the data obtained indicated the most pronounced increase in ecological risk in Finland and the Baltic countries.

#### Pesticide leaching to groundwater and occurrence in soil

The Danish pesticide leaching assessment program uses results from controlled leaching experiments at fixed field study sites, and up to 2014 it assembled data on 50 pesticides (Brüsch et al. 2015). Of these 50, only 16 showed no leaching at all and 18 were identified as pesticides of concern. Also, 16 of the 50 pesticides were observed to be leached at concentrations >0.1 μg/L, and this group included the herbicides metribuzin, rimsulfuron, terbuthylazine, and bifenox, and the fungicide metalaxyl-M. The leaching assessment results also indicate challenges regarding strongly sorbing pesticides that are mainly transported through macropores (e.g., glyphosate and diflufenican) and with respect to the long-term leaching of pesticide metabolites (e.g., metalaxyl metabolite CGA 108906, fluazifop-p-butyl metabolite TFMP, desamino-diketo, and diketo-metribuzin).

The thorough and consistent assessment of pesticide leaching that is being conducted in Denmark should be supplemented by comparable results from the other countries in the northern zone to enable a zonal evaluation of the pesticides of concern. Current soil monitoring results from Estonia show that the most frequently detected residues are tebuconazole and epoxiconazole for fungicides, trifluaraline and glyphosate for herbicides, and DDT (banned in 1997) for insecticides.

In Norway, data on pesticides in groundwater are very limited. However, screening sampling has revealed occurrence of pesticides in local drinking water wells in shallow groundwater systems beneath agricultural land (Roseth 2013), with the herbicides bentazon, atrazine (banned), and MCPA, the fungicide metalaxyl, and the metabolite BAM detected most often, in some cases at levels of >0.1 μg/L. A thorough assessment of pesticides revealed residues of several pesticides in groundwater in an area with fluvial deposits of sand with a top layer of silt and sandy silt and intensive cultivation of potatoes and cereals (Kværner et al. 2014); the herbicides bentazon and MCPA, the fungicide metalaxyl, and the metabolites ETU and BAM were found at concentrations above 0.1 μg/L.

In Latvia, no research is currently being conducted to address leaching of pesticides from agricultural areas into waterways and soil. This situation exists despite the fact that the most intensive farming is done in areas with soils that are vulnerable to leaching, and half of the national cereal and rape seed yield is obtained in these areas (Jansons et al. 2011). Furthermore, pesticide use statistics illustrate the central role of the herbicides employed in agricultural production in Latvia and also underline the need for further studies of the environmental fate of these substances under the conditions prevailing in this country (Central Statistical Bureau of Latvia 2015).

In Finland, the pesticides most frequently detected in groundwater have been triazine herbicides and their metabolites, and the dichlobenil metabolite BAM. Some drinking water sources have been closed during the last decade, because pesticide concentrations have exceeded the regulatory limits (0.1 μg/L for single substances or 0.5 μg/L for the sum of all pesticides).

#### Pesticide transport through runoff and drainage to surface water

In Sweden, surface water monitoring data show that herbicides are found more frequently than fungicides and insecticides (Lindström et al. 2015). The substances that exceeded the water quality objectives most often during the period 2002–2012 included the following: diflufenican, picoxystrobin (fungicide), isoproturon, MCPA, terbuthylazin (banned), metribuzin, metazachlor, tiacloprid (insecticide), and imidacloprid (insecticide).

Pesticide use statistics and monitoring in Estonia also suggest that herbicides constitute the most relevant group of compounds for in-depth studies. Pesticide use in Estonian agriculture is dominated by herbicides, with glyphosate and MCPA being the most widely applied, representing about 65 and 20 %, respectively, of the total sales of herbicide active ingredients (Statistics Estonia 2012). The Estonian monitoring results show that pesticides in general have seldom been found in surface and groundwaters, but glyphosate and AMPA have been detected on several occasions.

Pesticide monitoring in Denmark also includes a program focused on the aquatic environment (NOVANA) (Boutrup et al. 2015), which includes groundwater wells, surface and marine waters, and precipitation. Since the adoption of the Water Framework Directive (2000/60/EC), this program has mainly targeted the defined priority substances and to a much lesser degree analyzed pesticides that are currently in use. The herbicides BAM (metabolite of the banned herbicide diclobenil), glyphosate, and MCPA are the substances detected most frequently in stream water (Boutrup et al. 2015).

In Finland, the number of compounds detected has increased with an increasing number of compounds analyzed (from 98 to 218 over the period 2004–2014). MCPA and dichlorprop-P are the pesticides found most often in surface water monitoring, although the levels recorded have not been higher than the EQS values. However, concentrations of some low-dose sulfonylureas (e.g., triasulfurons) and some old and banned insecticides have more frequently exceeded their suggested EQS values (e.g., Karjalainen et al. 2014).

Monitoring results (1995–2012) from small agricultural streams in Norway show that the mobile herbicides MCPA and bentazone are the substances found most frequently, but the herbicide metribuzin is most often detected above concentrations that might have a negative effect on aquatic organisms (Stenrød 2015). This monitoring also demonstrates intensified use of fungicides (e.g., prothioconazole to control *Fusarium* head blight in cereal), as well more frequent detections of these substances, especially in years with increased pressure from fungal diseases and frequent intense rainfall events during the spraying season. Potential problems connected with insecticide use are difficult to discern by monitoring, because the limits of quantification for the analytical methods applied are often 100-fold higher than the P(N)ECs in the aquatic environment. Nevertheless, some challenges have been observed to be connected with use of the insecticide imidacloprid for coating potato tubers (Stenrød 2015) and in greenhouse production (Roseth 2012).

Increasing the area under low-tillage practices leads to more widespread use of the herbicide glyphosate to control weeds, but this substance is not included in all the stream water-monitoring programs in the northern zone. Monitoring results for the autumn and winter periods are especially scarce, which is particularly disturbing considering that glyphosate is applied on autumn stubble fields in cereal cropping.

This brief overview shows that the northern zone collaboration will enable identification of pesticides of concern in several environmental compartments (e.g., groundwater, surface water, and soil), as well as in the various agricultural practices. However, these observations also illustrate the need for increased harmonization of monitoring programs to facilitate comparison of results and also to pinpoint common pesticides of concern within the zone.

#### Pesticides in sediments

The monitoring programs in the northern zone are focused predominantly on pesticides found in water samples, which by nature are hydrophilic substances, and much less interest is given to hydrophobic pesticides sorbed to soils and sediments. In Sweden (Lindström et al. 2015) and in Finland (unpublished data), fewer compounds have been analyzed in sediment samples than in water samples, and the number of compounds actually detected is even lower. Most herbicides have sorption coefficients (Koc) well below 400 mL/g, but most fungicides and insecticides have Koc values above 400 and are more or less hydrophobic. It is possible that pesticides sorbed to soil particles and sediments are more persistent in the northern zone due to the cold climates, and this issue should be given further attention.

### Knowledge gaps and proposed measures

#### Define northern zone conditions to improve fate and risk assessment of pesticides

**Fig. 2:**
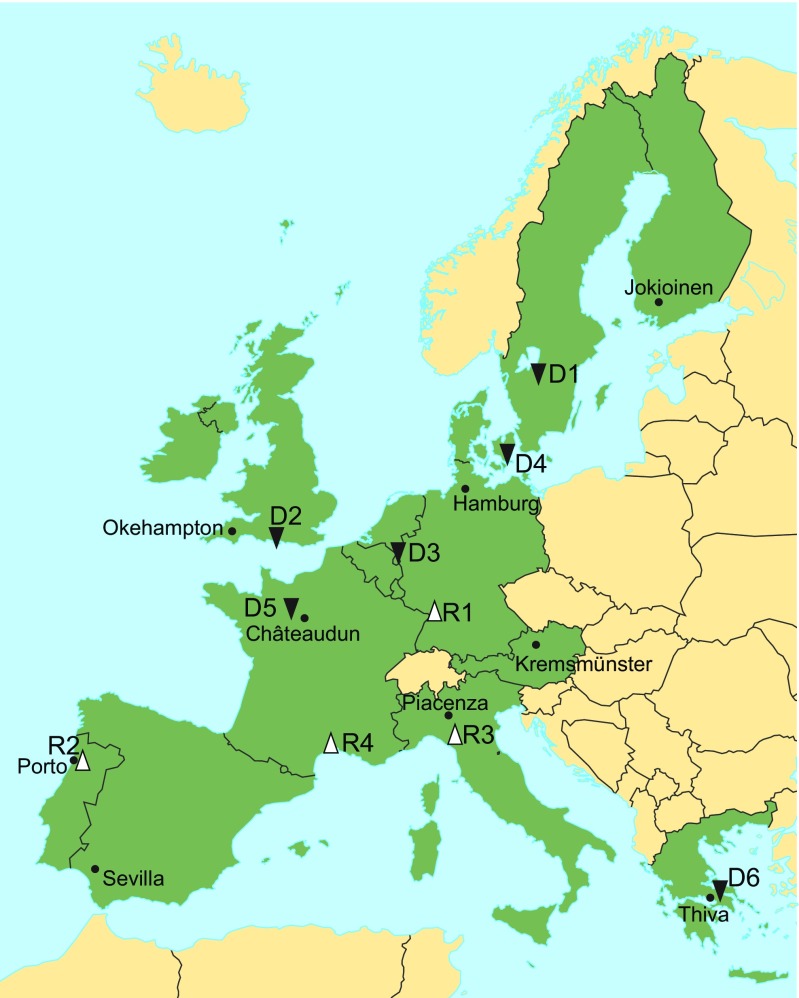
Location of FOCUS groundwater (*bullet*) and surface water (*black down-pointing triangle* = drainage, *increment* = runoff) scenarios. Adapted from FOCUS 2000, 2001

The division of Europe into a southern, a central, and a northern zone under the current pesticide regulations has advantages (e.g., a more efficient approval process), but unfortunately it also has some drawbacks. The development and coordination of pesticide risk assessment procedures for the EU and associated countries under the former EU pesticide regulations (Council Directive 91/414/EEC) resulted in a range of selected and recommended models and scenarios (Fig. 2) (FOCUS 2000, 2001). However, inasmuch as both the pesticide industry and European agriculture are to a large extent located in the countries in the central and southern zones, it is plausible that the specific conditions in the northern zone are not sufficiently represented in the common EU models and scenarios to allow adequate pesticide risk assessment in the northern zone countries.

More work is needed to confirm the capacity of the current FOCUS scenarios for assessing pesticide fate in the northern zone countries. To accomplish this task, it will be necessary to explicitly identify the aspects that define the northern zone with respect to the environmental factors that govern the fate of pesticides in the environment (e.g., temperature, light, rainfall intensity, snow/frost, and soil), and to evaluate this definition in comparison with the currently employed EU scenarios and endpoints. Within the northern zone, there is considerable diversity in soil and weather conditions, as well as in the predominant cropping practices and environmental concerns related to agricultural activities. Previous attempts to specify Nordic reference soils (Tiberg 1998) resulted in the selection and description of 13 different soils from Denmark, Finland, Norway, and Sweden, which were assumed to represent the following: (1) soils covering a large fraction of the Nordic area; (2) soils from the different climatic regions; and (3) environmentally sensitive soils. These efforts require revision and should include the Baltic countries to ensure that proper consideration is given to the common and varying soil properties within the northern zone. In the context of estimating the fate of crop protection chemicals, the focus should be on representative soil scenarios for the agricultural area in the northern zone countries.

There are several collaborative activities that should be initiated to improve and harmonize fate and risk assessment of pesticides in the northern zone: (1) re-evaluate existing data from field experiments focusing on usability in a regulatory context; (2) improve existing models and increase their usability for assessments of multiple metabolites, low application rates, and the effects of winter-related processes (e.g., soil freezing and thawing); (3) develop harmonized FOCUS surface runoff and groundwater leaching scenarios adapted to northern zone conditions; (4) investigate the suitability of EQS values as a measure of toxicity measures and, when possible, establish harmonized EQS values for pesticides and metabolites in the northern zone; (5) define the environmental conditions in the northern zone and analyze the representativeness of European (central/southern zone) conditions and modeling parameters.

#### Mitigation measures in the northern zone countries

The use of available risk mitigation measures should be increased within all the northern zone countries, and this should include technical approaches such as drift-reducing and other mitigation strategies (e.g., Reichenberger et al. 2007; Arvidsson et al. 2011). Pesticide runoff mitigation achieved with vegetated buffer strips is generally considered effective (Syversen and Bechmann 2004; Arora et al. 2010; Dunn et al. 2011), although the impact of this approach has been shown to vary greatly (Reichenberger et al. 2007), assumingly due to site-specific conditions. Topography influences the transport of pesticides from soil to water, and there is a lack of knowledge regarding how this affects the efficacy of measures such as grassed buffer strips (Tang et al. 2012). This issue is important in countries like Norway that has a large proportion of agricultural areas on relatively steep slopes, where use of buffer strips might be less successful than in flat areas. Thus, more detailed studies are needed to identify any similarities and differences within the zone, and these investigations should include modeling efforts to assess the efficacy of vegetated filter strips in the northern zone countries (i.e., VFSMOD; Muñoz-Carpena et al. 1999).

According to monitoring results, detections of pesticide residues in the environment largely reflect use patterns, which underlines the importance of good agricultural practices (e.g., dosage and timing). Hence, guidance tools and stewardship represent further essential approaches to increase the sustainability of pesticide use by raising awareness among farmers. Existing web-based resources designed to promote integrated pest management (IPM) and sustainable pesticide use in the northern zone include the following: the Swedish online guidance tool aimed at reducing the environmental impacts from agriculture (*Greppa Näringen*; www.greppa.nu), with crop protection and pesticide use as central topics; the Norwegian forecast system for plant diseases, pests, and weeds (VIPS; www.vips-landbruk.no); the Danish decision support system for weed management (*Planteværn* Online; ipmdss.dk); the Finnish online information about crop protection and forecasts of the occurrence of selected pests (*Kasper*; https://portal.mtt.fi/portal/page/portal/kasper). To establish IPM methods adapted to northern zone conditions, efforts should be made to further develop the collaboration between the countries in this zone to achieve improved pest forecasting, precision farming tools, and decision support systems for integrated pest management and sustainable pesticide use.

## Conclusions, recommendations, and outlook

There is a need to improve and harmonize fate and risk assessment of pesticides in the northern zone using information derived from knowledge exchange between researchers. The Nordic-Baltic Pesticide Fate Workshop highlighted the many possibilities that are within reach. The main conclusion drawn from the workshop discussions was the need to identify the specific environmental conditions in the northern zone and to ascertain how this picture can be harmonized in (regulatory) pesticide fate modeling. Both the weather conditions (e.g., temperature, light, rainfall intensity, snow/frost) and soil conditions (e.g., soil type, freezing/thawing of soil) vary markedly between different areas within the northern zone. Furthermore, the agricultural practices in the Nordic and Baltic countries differ markedly, being influenced not only by topography and soil and weather conditions, but also by sociocultural conditions and political decisions. Successful research and regulatory collaboration within the northern zone is essential to ensure that the specific conditions in this region are adequately addressed under the current European pesticide regulations. The Nordic-Baltic pesticide fate network should propose collaborative projects that entail regulatory monitoring and research to achieve the following:

- Define northern zone environmental conditions and assess the representativeness of European (i.e., central and southern zones) conditions and modeling parameters.
- Improve existing models and increase their usability in assessing multiple metabolites, low application rates, and the effects of winter-related processes (soil freezing and thawing).
- Review existing data from field experiments to assess usability in a regulatory context.
- Develop harmonized FOCUS surface runoff and groundwater leaching scenarios adapted to northern zone conditions.
- Determine the suitability of EQS values as a measure of toxicity and develop harmonized EQS values for pesticides and metabolites in the northern zone.
- Establish a joint web information platform to facilitate dissemination and exchange of research and regulatory knowledge on pesticides and the fate of these substances in the northern zone.

It is imperative to ensure a continued focus on these issues attained through the regulatory northern zone collaboration. Furthermore, the Nordic-Baltic pesticide fate network should propose a procedure outlining how pesticides of concern within the zone are to be identified and to be subjected to further actions by the northern zone regulatory work group. This procedure should include monitoring of chemical status and biodiversity (surface water) and also assessment of leaching (unsaturated zone, drainage, and groundwater). These tasks should be conducted on a selection of farms after approval of new pesticides and theoretical toxicity analysis, followed by more specific laboratory and field studies and risk assessment by modeling after identifying pesticides of concern. There must be agreement regarding both threshold values (surface and groundwater concentrations and toxicity assessment results) and the frequency of surpassing defined thresholds that can be accepted as qualification for more detailed studies.
